# An Unusual His/Asp Dyad Operates Catalysis in Agar-Degrading Glycosidases

**DOI:** 10.1021/acscatal.4c04139

**Published:** 2024-11-01

**Authors:** Mert Sagiroglugil, Alba Nin-Hill, Elizabeth Ficko-Blean, Carme Rovira

**Affiliations:** † Departament de Química Inorgànica i Orgànica & IQTCUB, Universitat de Barcelona, Martí i Franquès 1, Barcelona 08028, Spain; ‡ Laboratory of Integrative Biology of Marine Models, Station Biologique de Roscoff, CNRS, Sorbonne Université, UMR8227, Roscoff 29688, France; § Institució Catalana de Recerca i Estudis Avançats (ICREA), Passeig Lluís Companys, 23, Barcelona 08020, Spain

**Keywords:** agarose, 3,6-anhydro-l-galactosidase, carbohydrate-active enzymes, metadynamics, molecular dynamics, quantum mechanics/molecular mechanics, catalytic mechanism

## Abstract

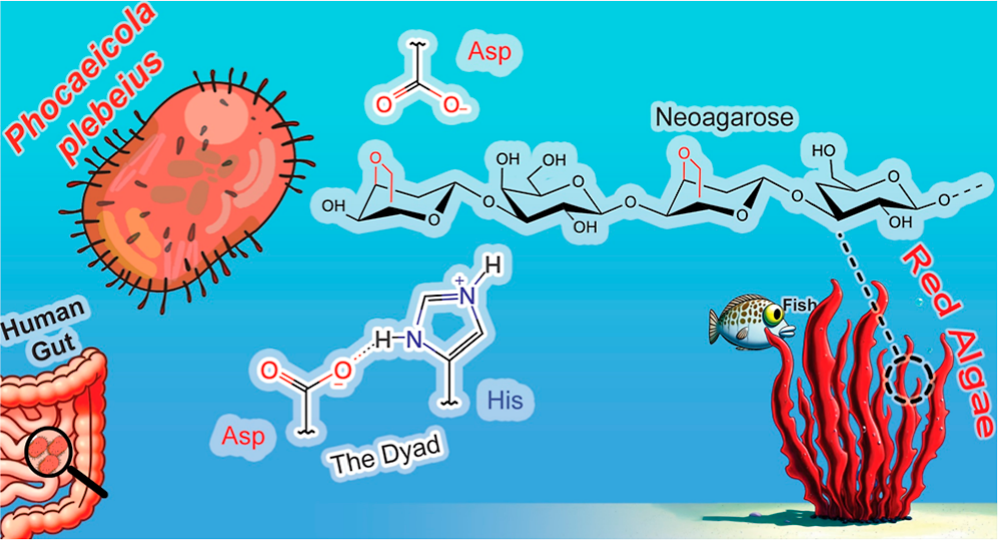

Agarose motifs, found in agars present in the cell walls of red algae, consist of alternating units of d-galactose (G) and α-3,6-anhydro-l-galactose (LA). Glycoside hydrolases from family 117 (GH117) cleave the terminal α-1,3-glycosidic bonds, releasing LA units. Structural studies have suggested that these enzymes use unconventional catalytic machinery, involving a histidine (His302) as a general acid rather than a carboxylic residue as in most glycosidases. By means of quantum mechanics/molecular mechanics metadynamics, we investigated the reaction mechanism of *Phocaeicola plebeius* GH117, confirming the catalytic role of His302. This residue shares a proton with a neighbor aspartate residue (Asp320), forming a His/Asp dyad. Our study also reveals that, even though the sugar unit at the *–1* subsite (LA) can adopt two conformations, ^4^*C*_1_ and ^1,4^*B*, only the latter is catalytically competent, defining a ^1,4^*B* → [^4^*E*]^‡^ → ^1,4^*B* (→ ^4^*C*_1_) conformational itinerary. This mechanism may be applicable to similar enzymes with a His/Asp dyad in their active sites, such as GH3 β-*N*-acetylglucosaminidase and GH156 sialidase. These insights enhance our understanding of glycosidase catalytic strategies and could inform the engineering of enzymes for the more efficient processing of seaweed.

## Introduction

**Figure 1:**
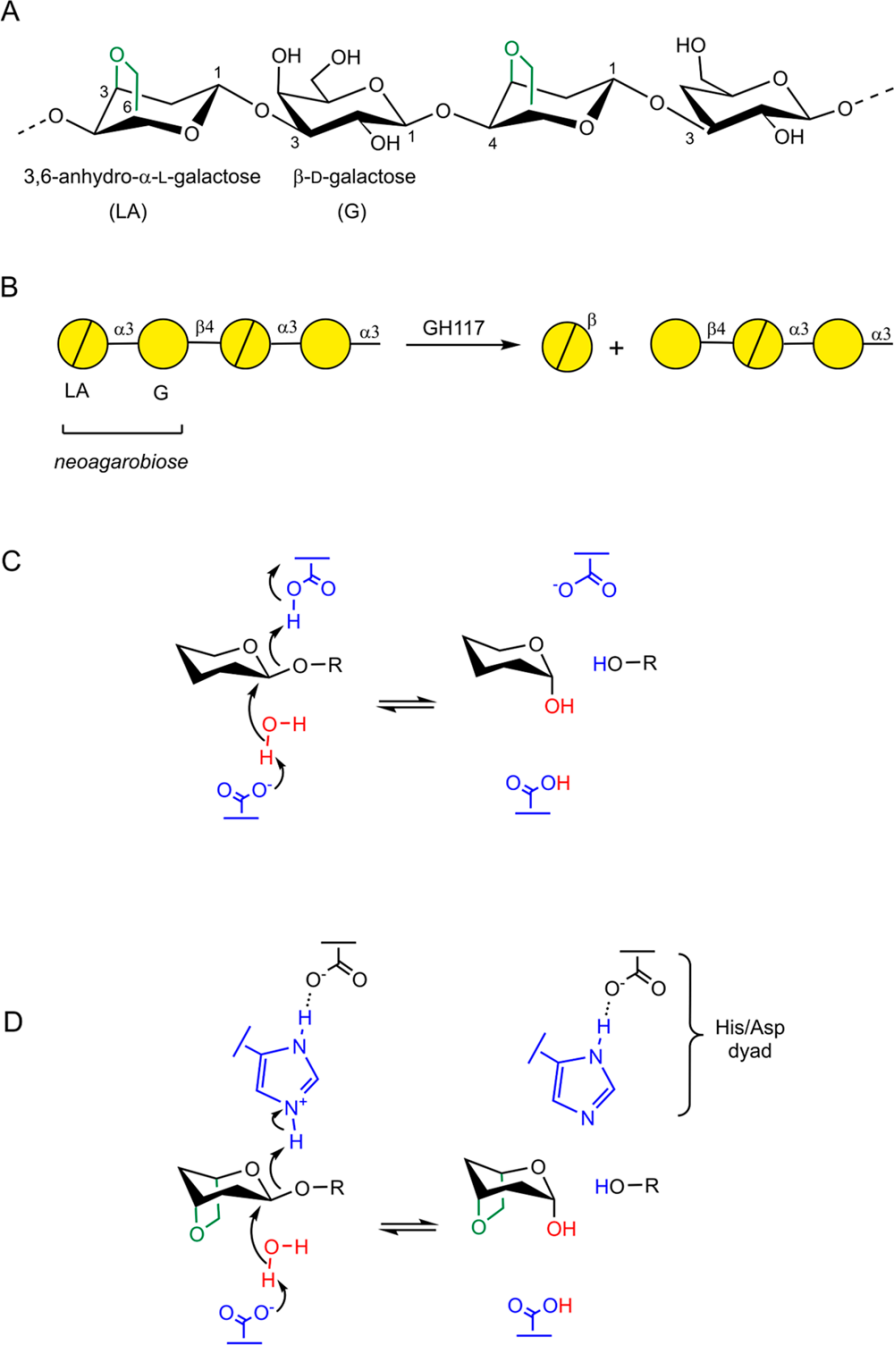
(A) Chemical structure of agarose, a linear polysaccharide made up of d-galactopyranose and 3,6-anhydro-l-galactopyranose. (B) Reaction catalyzed by *Pp*GH117. The enzyme acts on neoagaro-oligosaccharides once the β-1,4 bonds have been cleaved by endohydrolases. (C) Classical reaction mechanism for inverting glycosidases. (D) Reaction mechanism that has been proposed for *Pp*GH117 from X-ray crystallography studies.

Agarose is a linear polysaccharide commonly found in agarophyte red algae. It is composed of longer chains of alternating bicyclic α-(1,3)-3,6-anhydro-l-galactose (LA) and β-(1,4)-d-galactose (G) units (also known as neoagarobiose) (Figure 1).^1^ The bridged structure of LA residues allows for the formation of organized helical structures, resulting in the creation of high-strength gels that are resistant to degradation. This makes them useful for a variety of applications, including microbiological, molecular biological, and food-related uses.^2^

The important role that red macroalgae play as primary producers in marine ecosystems has raised interest in the enzymes involved in the degradation and metabolization of algal carbohydrates.^2b,3^ The enzyme α-(1,3)-3,6-anhydro-l-galactosidase^4^ is a member of the family 117 glycoside hydrolases (GHs) (GH117)^5^ and is found in *Phocaeicola plebeius* (formerly *Bacteroides plebeius*), a bacterium in the human gut microbiota.^6^ This exo-acting enzyme, coded in a porphyran PUL,^7^ has the ability to remove the LA residue from the non-reducing end of neoagaro-oligosaccharides (Figure 1B), cleaving the α-1,3 glycosidic bond between LA and G (LA–G or neoagarobiose) with inversion of configuration.^8^ GH117 enzymes tolerate neooligosaccharides of different lengths.^4,8^ In the particular case of *Pp*GH117 (formerly *Bp*GH117), the enzyme is active on neoagaro-oligosaccharides, showing significant activity on neoagarobiose but also demonstrating activity on neoagarotetraose and neoagarohexaose.^9^ Therefore, *Pp*GH117 is an important player in the degradation and utilization of red algal agars.^7^

**Figure 2:**
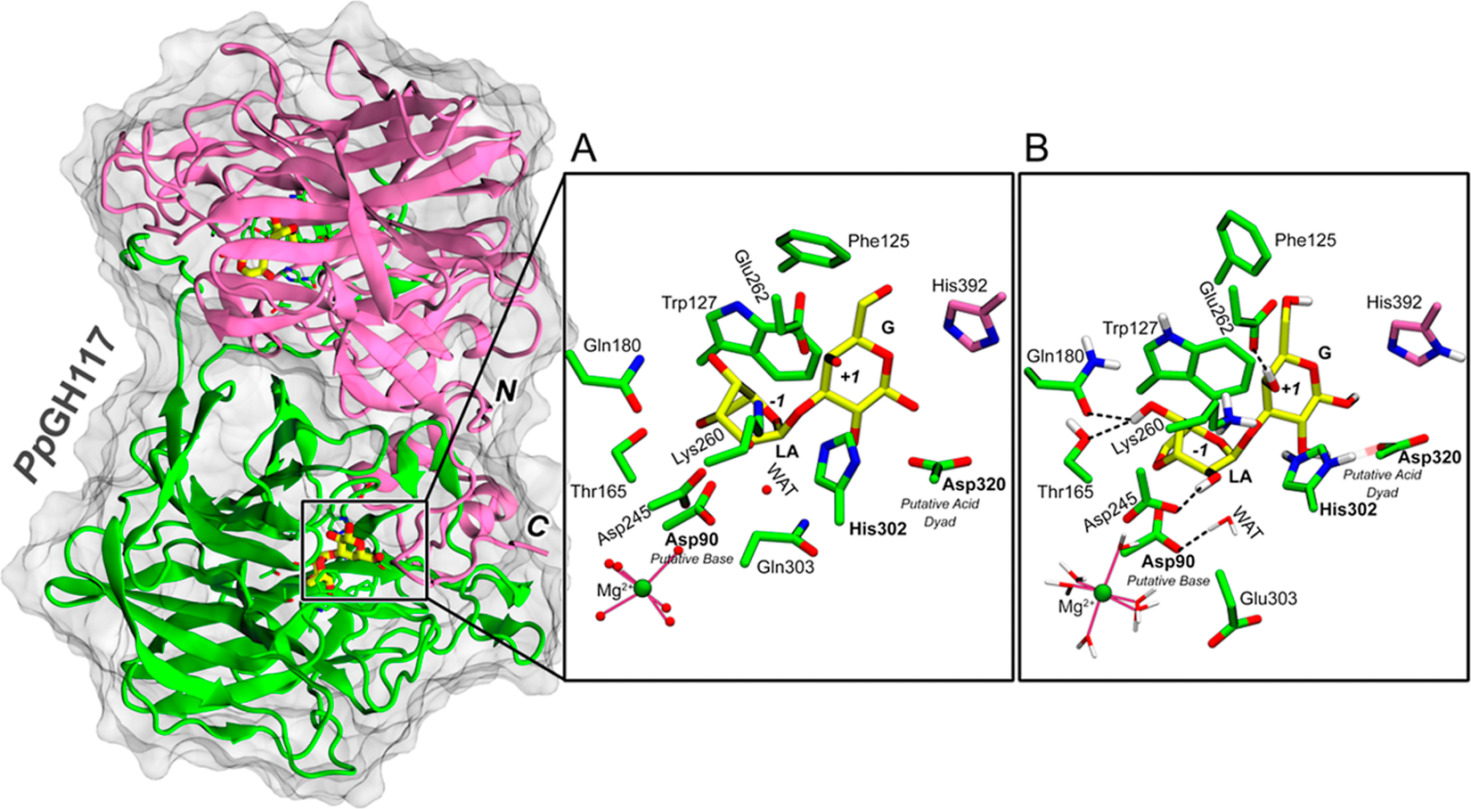
(A) X-ray structure of *Pp*GH117_Glu303Gln in complex with neoagarobiose (LA–G) (PDB 4AK7). The closest residues to the neoagarobiose substrate of one of the protein subunits are shown. (B) Active site structure obtained from QM/MM MD simulations. Hydrogen atoms attached to C atoms have been omitted for clarity.

*Pp*GH117 is a dimeric enzyme in which the N-terminal residues of one monomer form a helix-turn-helix domain that interacts with the other monomer to stabilize the dimer (Figure 2). The C-termini of each monomer are located at the opening of the active site of the opposite monomer, narrowing the entrance to the active site.^8^ A Michaelis complex structure of a *Pp*GH117 3,6-anhydro-l-galactosidase mutant (*Pp*GH117_Glu303Gln) in complex with its natural substrate neoagarobiose was solved by Hehemann et al. in 2012.^9^ A product complex was subsequently solved in a marine bacterium, confirming the inverting mechanism.^8^ It was suggested that Asp90 and His302 may function as a general base and general acid, respectively, in the catalytic reaction (Figure 1D).^9^ This is different from the type of catalytic residues found in most inverting GHs, in which a pair of carboxylic acid–based residues (Asp or Glu) usually play this role (Figure 1C).^10^ These differences raise the question of whether the catalytic mechanism of *Pp*GH117 differs from the Koshland single-displacement mechanism of most inverting GHs (Figure 1C).

The structure of *Pp*GH117_Glu303Gln in complex with neoagarobiose revealed that His302 closely interacts with residue Asp320. It was hypothesized that this residue stabilizes the positive charge on the His at the Michaelis complex.^9^ In addition, the active site contains a glutamic acid residue (Glu303, mutated to Gln in the crystal structure) near the glycosidic oxygen (Figure 2A) that could act as a catalytic acid. Unfortunately, mutagenesis studies could not unambiguously assess the role of His302 or Glu303 in catalysis as all single mutations of critical residues (Glu303, Asp90, Asp245, Glu167, and His302) inactivate the enzyme.^3^ It was also proposed that Glu303 may have the role of regulating p*K*_a_ of the putative general base Asp90.

An important aspect of GH catalysis is the conformational itinerary that the substrate follows during catalysis, which can guide inhibitor design.^11^ The conformational itinerary can be inferred based on a suitable structure of the Michaelis complex, which can inform quantum mechanics/molecular mechanics (QM/MM) simulations of the reaction mechanism.^12^ Based on the Michaelis complex structure of a *Pp*GH117 mutant with neoagarobiose, Hehemann et al. reported that the sugar ring at the *–1* subsite (hereafter referred to as the ″*–1* sugar”) adopts a *B*_1,4_ conformation.^9^ However, analysis of the corresponding crystal structure shows that actually the *–1* sugar was refined in the “opposite” ^1,4^*B* conformation, in consistency with the most favored conformation of a 3,6-anhydro-l-galactose molecule.^9^ It is to be noted that, while a *B*_1,4_ conformation would be possible for 3,6-anhydro-d-galactose (d enantiomer),^13^ it is not allowed for 3,6-anhydro-l-galactose (l enantiomer). Because of the steric constraints of the 3,6-anhydro bridge, 3,6-anhydro-l-galactose predominantly prefers a conformation where the C4 atom is placed above the main sugar plane, such as ^4^*C*_1_, ^4^*E*, or ^1,4^*B* (Figure S1).

Guided by the structure of *Pp*GH117_Glu303Gln in complex with neoagarobiose,^9^ we investigated the enzyme reaction mechanism using QM/MM methods, with the objective of assessing whether a histidine residue can serve as general acid and provide a detailed atomistic view of the reaction coordinate. We found that the His302/Asp320 pair acts as a general acid dyad, facilitating catalysis via a low-energy-barrier hydrogen bond. We also confirm the role of Asp90 as a general base and predict that the enzyme follows a ^1,4^*B* → [^4^*E*]^‡^ → ^1,4^*B* conformational itinerary during catalysis, with subsequent relaxation toward a ^4^*C*_1_ conformation once the aglycon leaves the active site.

## Results and Discussion

### Michaelis Complex Structure and Dynamics

The crystal structure of *Pp*GH117_Glu303Gln with its natural substrate neoagarobiose (PDB 4AK7, at 1.80 Å resolution) was used to build an initial model for the simulations. The Glu303Gln mutation was reverted, and the system was equilibrated by classical molecular dynamics (MD) (3.0 μs) with FF14SB,^14^ GLYCAM,^15^ and TIP3P^16^ force fields. During the simulation, the sugar at the *–1* subsite (LA) adopted a ^4^*C*_1_ conformation. However, a word of caution is necessary since classical force fields often fail to reproduce conformations of pyranose rings.^12,17^ Therefore, another MD simulation was performed in which the sugar conformation was restrained relative to that of the X-ray structure (^1,4^*B*). Subsequent QM/MM metadynamics simulations (see the next section) confirmed that both conformations are stable minima, with ^1,4^*B* being the most stable.

The classical MD simulations also showed that the active site water molecule (WAT in Figure 2B) maintains a persistent interaction with Asp90, the putative general base. The water molecule is also close to the anomeric carbon, in place for catalysis. It is to be noted that Asp90 is in the vicinity of the Mg^2+^ coordination sphere. However, it does not interact with it, unlike the general base of certain GHs (e.g., GH38 and GH92).^18^ The metal ion in GH117, being far from the catalytic water (5.9–7.8 Å in our simulations) and located in a solvent channel, is likely to act as a water reservoir, delivering the water molecules to the active site when needed.^4^

The MD simulations also show that active site His302 forms a hydrogen bond with the glycosidic oxygen via its N_ε_–H proton, while its N_δ_ atom interacts with Asp320, forming the putative catalytic dyad. Therefore, the three residues, Asp90 (putative base), Asp320, and His302 (putative acid), are properly positioned for the chemical reaction. The alternative catalytic base, Glu303, adopts a different orientation than in the X-ray structure. This is not surprising in view of the Glu303Gln mutation in the latter. Nevertheless, Glu303 can also form a hydrogen bond interaction with the active site water molecule if the *–1* sugar is in a ^4^*C*_1_ conformation (Figure S2), indicating a possible competition between Glu303 and Asp90 to abstract a proton from the water molecule. These three active site residues, histidine, aspartic acid, and glutamic acid, are conserved throughout the GH117 family, with only two exceptions.^8^ Thus, we cannot fully exclude the participation of either Asp90 or Glu303 as a general base in catalysis based on analysis of the Michaelis complex alone.

### Substrate Conformational Free Energy Landscape

To gain further insights into the preferred conformation of the *–1* sugar (LA) in the active site of *Pp*GH117, we turned to QM/MM simulations. One representative snapshot of the MD trajectory in which the system is in a reactive configuration was selected for QM/MM MD simulations (20 ps). The simulations show that the active site exhibits the same configuration as in the previous classical MD simulations, but the N_δ_–H proton of His302 is practically shared with Asp320 (the N_δ_–H distance is 1.32 ± 0.21 Å; the N_δ_–O_Asp320_ distance is 2.54 ± 0.09 Å), forming a low-barrier hydrogen bond. This suggests that His302, in conjunction with Asp320, could play a role in catalysis.

**Figure 3:**
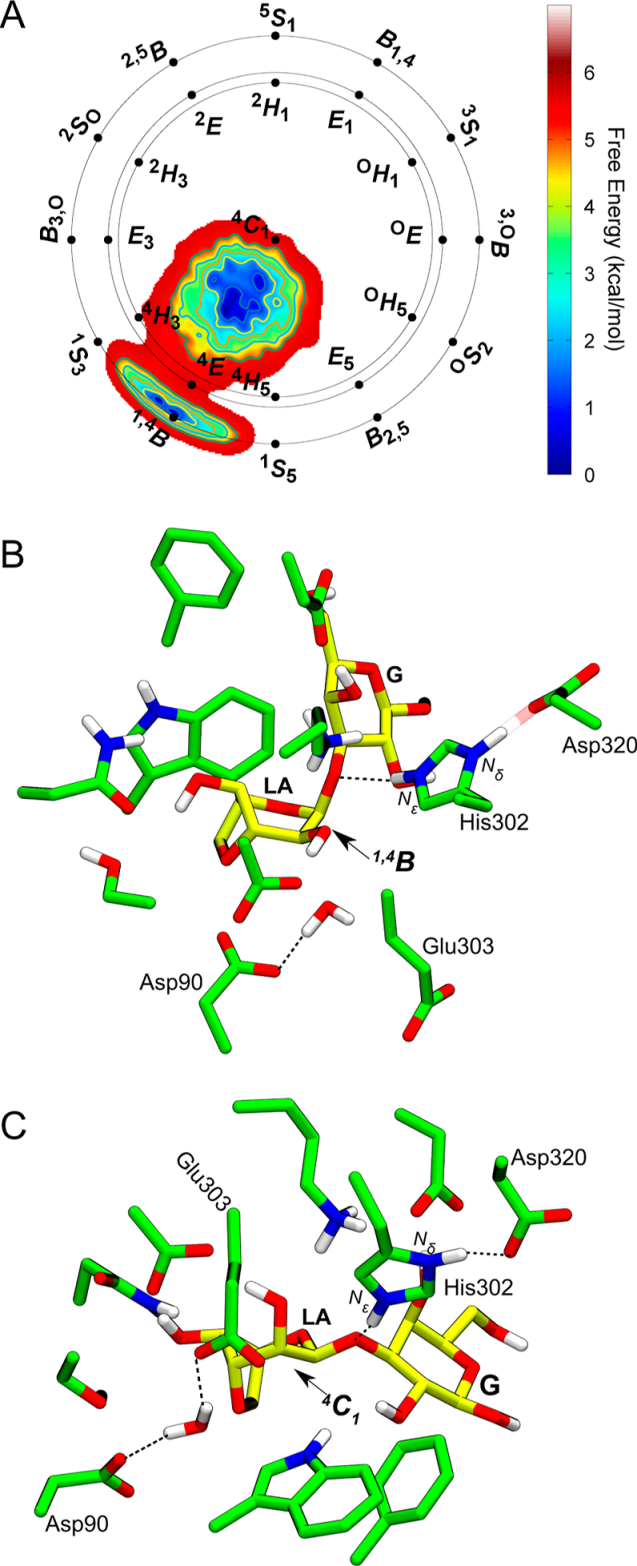
(A) Conformational FEL of the LA sugar in the active site of *Pp*GH117. The Northern hemisphere of Cremer–Pople sphere^11,20^ is shown. Contour lines at 1 kcal mol^–1^. (B) Close view of the active site with the LA sugar in the ^1,4^*B* conformation, corresponding to the most stable minimum of the conformational FEL. (C) Active site with the LA sugar in the ^4^*C*_1_ conformation. Different views have been selected in (B) and (C) to facilitate comparison of the leaving group orientation in each case.

The metadynamics approach,^19^ along with Cremer–Pople ring puckering coordinates^20^ as collective variables (CVs), was used to compute the free energy landscape (FEL) of the *–1* sugar (LA) in the active site. This approach has been previously proven to be very efficient to identify stable substrate conformations in GHs.^12,21^ The computed FEL (Figure 3A) shows that only two conformations are possible in the *Pp*GH117 active site: ^4^*C*_1_ and ^1,4^*B*. The latter conformation is favored by ≈ 2 kcal mol^–1^ over the former, in consistency with the conformation observed in the X-ray structure of *Pp*GH117_Glu303Gln in complex with neoagarobiose.

A similar scenario was observed in the absence of the enzyme (Figure S3), with the boat conformation being just 1 kcal mol^–1^ lower in energy with respect to the chair. Unlike what was previously found on most GHs,^12^ the enzyme does not seem to change significantly the conformational landscape of *–1* sugar with respect to that isolated substrate. This is probably due to the steric constraints of the 3,6-anhydro bridge.

Notably, the boat conformation has the galactoside leaving group in an axial orientation, ready for the nucleophilic (S_N_2) attack of the water molecule from the opposite face of the LA sugar (Figure 3B). In contrast, the leaving group is in a less suitable (equatorial) orientation when the LA is in the ^4^*C*_1_ conformation (Figure 3C). Therefore, only the most stable ^1,4^*B* conformation of the LA is expected to be catalytically competent.

### Enzyme Reaction Mechanism

**Figure 4:**
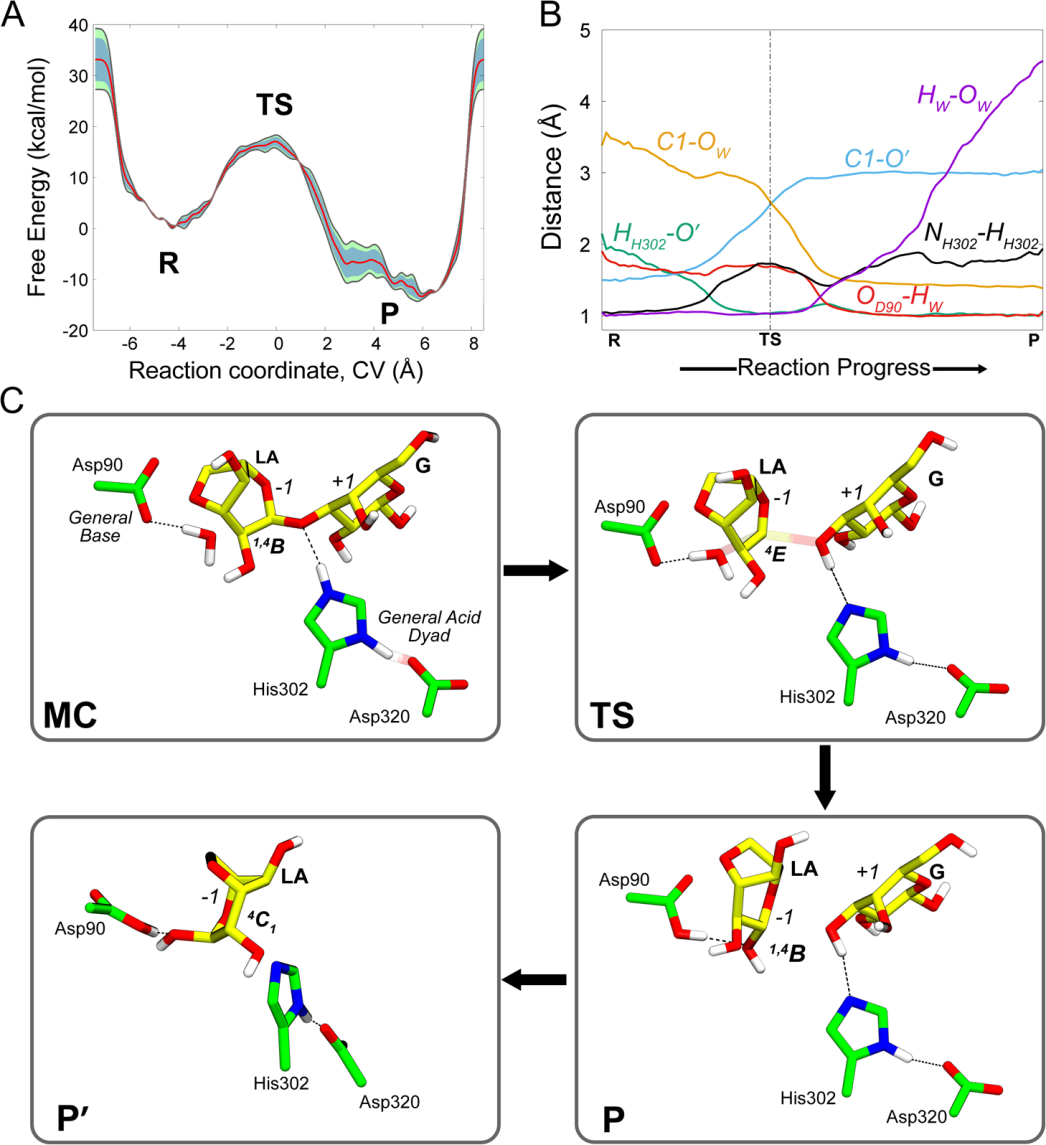
(A) Free energy profile of the reaction catalyzed by *Pp*GH117 obtained from QM/MM metadynamics simulations. Gray curves represent free energy profiles from two transition cycles, respectively, whereas the red curve is the exponential average of these profiles. Color-filled regions indicate the standard deviation (green) and the standard error (blue). (B) Evolution of the main catalytic distances along the normalized reaction coordinate. (C) Representative structures of the main states along the reaction coordinate (MC, TS, and P). Note that P′ was obtained upon additional unbiased QM/MM MD simulations removing the leaving group sugar. Figure S5 shows a magnified view around the P state, highlighting the finer variations shown in (B). Hydrogen atoms attached to C atoms have been omitted for clarity.

To investigate the enzyme reaction mechanism, we performed QM/MM metadynamics simulations^22^ starting from a configuration in which the substrate is in the most stable ^1,4^*B* conformation (Figure 3). One CV, combining the main distances expected to be broken or formed during the reaction, was used (Figure S4). The distance between Asp90 and the putative catalytic water was included in the CV as this residue is the most likely residue to play the role of a catalytic base. The reaction free energy profile obtained from the simulation (Figure 4A) shows a concerted reaction [with only one transition state (TS)] with an energy barrier of 16.8 ± 1.3 kcal mol^–1^ and is exergonic. These results are in very good agreement with the energy barrier estimated from experimental kinetic data (15.60 and 15.89 kcal mol^–1^ for two GH117 enzymes from *Cellvibrio sp.*).^23^ This indicates that the reaction involving Asp90 and His302 as catalytic bases and acids, respectively, is feasible.

The computed reaction pathway shows that the chemical reaction begins with elongation of the glycosidic bond, while the leaving group retains its axial orientation, and the N_ε_–H proton of His302 forms a hydrogen bond with the glycosidic oxygen (Figure 4B,C). As the glycosidic bond elongates, the N_ε_–H proton transfers to the glycosidic oxygen, and the *–*1 sugar evolves toward an ^4^*E* conformation, reaching the reaction TS. The flattening of the sugar ring (C5–O–C1–C2 = 11°) and the shortening of the C1–O5 bond (1.41 ± 0.06 to 1.29 ± 0.01 Å) indicate the formation of an oxocarbenium ion-like species at the TS. Both the glycosidic bond and the bond between the water oxygen atom and the anomeric carbon are partially broken/formed, respectively. In particular, the C1–O′ increases from 1.5 Å at the MC to 2.6 Å at the TS, while the O_w_–C1 bond decreases from 3.5 to 2.6 A (Figure 4C). Lys260 gets closer to the glycosidic oxygen (Figure S11), stabilizing it. The proton of His302 that was shared with Asp320 in the MC fully moves to His302 at the TS (N_δ_–H = 1.06 ± 0.04 Å), and the His/Asp dyad is kept by a hydrogen bond (N_δ_–H···O_Asp_ = 1.70 ± 0.18 Å). This indicates that Asp320 assists leaving group protonation by modulating protonation/deprotonation of catalytic acid His302. After the TS, Asp90 fully deprotonates the water molecule, and the remaining hydroxyl group completes the nucleophillic attack on the anomeric carbon, resulting in an inversion of the anomeric configuration (P in Figure 4C).

It is interesting to note that the LA sugar features a distorted ^1,4^*B* conformation in the product state (P). This differs from the conformation observed in the closely related *Zg*GH117 enzyme in complex with β-3,6-anhydro-l-galactose (a product complex).^8^ Nevertheless, the crystal structure lacks the galactose leaving group, which is still present in our product complex. Additional QM/MM MD simulations upon removing the leaving group show that the *–*1 sugar (3,6-anhydro-l-galactose) evolves spontaneously toward a ^4^*C*_1_ conformation (P′ in Figure 4), in excellent agreement with the experimental structure.^8^

Finally, we considered exploring alternative catalytic strategies such as a pathway starting from an alternative conformation (^4^*C*_1_) of the *–1* sugar. To this aim, we applied the same protocol but starting from a structure in which the *–1* sugar adopts a ^4^*C*_1_ conformation. This is a stable minimum of the substrate conformational FEL (Figure 3) that should be partially populated at room temperature. In the ^4^*C*_1_ conformation, the catalytic water molecule is further away from the anomeric carbon (5.2 Å) compared to the ^1,4^*B* conformation (3.3 Å), as the 3,6-anhydro bridge exerts steric effects and impedes its approach to the sugar anomeric carbon. However, both Glu303 and Asp90 are in a suitable position to deprotonate it (Figure 3C). The catalytic acid dyad (His302/Asp320) is also well poised for the transfer of a proton to the glycosidic oxygen. Nevertheless, all our attempts to obtain a plausible mechanism, considering either Asp90 or Glu303 as the catalytic base, failed (the system reached a TS with a very high free energy barrier, ∼40 kcal mol^–1^, Figure S6) and/or had chemically unreasonable configurations (Figures S7 and S8). We think that this is due to two factors: the suboptimal position of the catalytic water and the fact that the α-linked G leaving group is in an equatorial orientation when the sugar is in the ^4^*C*_1_ conformation, making direct nucleophilic attack less efficient. We thus conclude that Glu303 cannot play the role of a catalytic base and that the ^4^*C*_1_ conformation is not catalytically productive.

## Conclusions

In this work, we have investigated the mechanism of *P. plebeius* 3,6-anhydro-l-galactosidase (*Pp*GH117) by means of QM/MM MD methods. Our simulations show that two conformations of the LA unit are possible in the enzyme active site (^4^*C*_1_ and ^1,4^*B*), but only one is catalytically productive (^1,4^*B*), which is also the conformation observed in the X-ray structure of the enzyme mutant in complex with neoagarobiose.^9^ In addition, our QM/MM metadynamics simulations show that the enzyme follows a “classical” S_N_2 mechanism, consisting of a concerted reaction with a dissociative TS, as usually found in glycosidases. The reaction involves an oxocarbenium ion-like TS in which the LA sugar adopts an ^4^*E* conformation, following a ^1,4^*B* → [^4^*E*]^‡^ → ^1,4^*B* (→ ^4^*C*_1_) catalytic itinerary. We also demonstrated that Asp90 is the catalytic general base rather than Glu303, and His302 is the catalytic general acid. Glu303 is likely to play a role as p*K*_a_ modulator of the environment, as suggested by Hehemann et al.^9^ His302 works in tandem with Asp320, sharing a proton with it that can shuttle from one residue to the other, as needed during the chemical reaction.

The mechanism of *Pp*GH117 involves an aspartate-stabilized histidine residue as a catalytic acid, together with an aspartate playing the role of a general base. Though not identified in all GH3 enzymes, this type of general acid catalytic dyad has been proposed for GH3 NagZ enzymes such as the β-*N*-acetylglucosaminidase from *Bacillus subtilis* (Asp232-His324 catalytic dyad), a retaining GH.^24^ Interestingly, it has also been proposed recently that a GH156 sialidase,^25^ which acts with inversion of configuration, involves a histidine as a general acid residue, based on structures of inhibitor and product complexes. Inspection of that structure shows that His is also interacting with an aspartate residue (His134–Asp132 dyad) similar to *Pp*GH117. Therefore, the results obtained for *Pp*GH117 3,6-anhydro-l-galactosidase can probably be extended to these His–Asp dyad GHs that, although differing in structure and substrate specificity, are also exo-acting GHs. These previous experimental investigations, together with the present computational results, highlight distinct strategies used by GHs to catalyze the hydrolysis of the glycosidic bond.

## Methods

### System Preparation

The starting point for the simulations was the X-ray structure of the *Pp*GH117_Glu303Gln dimer in complex with neoagarobiose (PDB ID 4AK7). Both subunits were used in the simulations since a His residue of one monomer interacts with the *+1* sugar of the other monomer. The Glu303Gln mutation was reverted, and the Ca^2+^ ion (anomalously present only in one protein chain) was replaced by a chlorine ion, as present in the opposite subunit. Both the crystal waters and ions (Mg^2+^ and Cl^–^) were retained. The enzyme was embedded in a cubic water box extending 10 Å away from the protein surface (40256 water molecules). The atomic partial charges (RESP) of neoagarobiose were calculated at the HF/6-31G* level of theory with Gaussian16.^26^ PKa values of enzyme residues were calculated using the H++ server (Table S1), and neutral pH values were used.^27^ The net charge of the system was neutralized by adding two sodium ions. The active site His302, expected to act as the general acid, was taken as doubly protonated, while Asp320, forming a dyad with it, and the putative general base Asp90 were both considered as deprotonated (see Figure S9 and Table S1).

### Classical MD Simulations

The GROMACS 2021.4 software^28^ was used to perform all the classical MD simulations. The enzyme and substrate were described with the force fields FF14SB^14^ and GLYCAM06,^15^ respectively, whereas the TIP3P water model^16^ was used for the water molecules. The input files, including the topology file, were generated using AmberTools22^29^ and subsequently converted into GROMACS-compatible formats with the ACPYPE software.^30^ The structure was first relaxed using the steepest descent algorithm and was subsequently equilibrated under NVT and NPT conditions, respectively. The V-Rescale^31^ thermostat was used to set the system temperature to 300 K during the NVT equilibration for 1 ns. During the NPT equilibration, both the V-Rescale thermostat and Parrinello–Rahman barostat^32^ were used to regulate the density of the solution for 1 ns. Five independent production MD runs (0.1 μs each) were performed. Additional production runs (5 × 0.5 μs) were performed with position restraints (where the force constants *k*_x_, *k*_y_, and *k*_z_ were set to 1000 kJ mol^–1^ nm^–2^) on the sugars to maintain the conformations of the crystal structure (Figure S10).

### QM/MM MD Simulations

QM/MM simulations were performed using the CP2K v9.1 software^33^ using two snapshots from the classical MD simulations, in which the LA sugar is either in ^4^*C*_1_ or ^1,4^*B* conformation. The complete substrate, the catalytic water, the side chain of the general base (Asp90), the side chains of the general acid dyad (His302 and Asp320), and the alternative putative catalytic base Glu303 were included in the QM region. The link atoms coupling the QM and MM regions were chosen as the C_β_ atoms of the QM residues. QM residue charges were redistributed to ensure a neutral charge for the MM region. The LJ parameters of the hydrogen atoms of the water molecules, as well as the hydroxyl group hydrogen atoms of Ser, Thr, and Tyr, were taken from the GAFF2 force field.^34^ The QM region was described by density functional theory using the Perdew–Burke–Ernzerhof functional,^35^ along with Goedecker–Teter–Hutter^36^ pseudopotentials. Triple-ζ valence polarized basis set functions^37^ were used to expand the Kohn–Sham orbitals, with a 350 Ry cutoff. The conjugate gradient method was used to optimize the structure of the snapshots that were selected from the classical MD trajectory, which were subsequently thermally re-equilibrated at 300 K for 20 ps, with an MD step size of 0.5 fs. All the QM/MM MD simulations were performed under NVT conditions using a V-Rescale thermostat^31^ with a coupling constant of 10 fs.

### QM/MM Metadynamics Simulations

The conformational landscape of the *–1* sugar was computed by using QM/MM metadynamics. Cartesian Cremer–Pople puckering coordinates^20 ,21^ divided by the ring puckering amplitude (*Q*)^21a^ (CV1 = *q*_x_/*Q*; CV2 = *q*_y_/*Q*; CV3 = *q*_z_/*Q*) were used as CVs, as in recent studies.^38^ Two structures from classical MD simulations were re-equilibrated with QM/MM MD, one having a relaxed ^4^*C*_1_ conformation and the other having a distorted ^1,4^*B* conformation. A QM/MM metadynamics simulation of sugar puckering was performed for each of these structures, using the PLUMED2 plug-in^39^ along with CP2K. The biasing parameters of the Gaussians were initially set to 1.0 kcal mol^–1^ in height, which was decreased to 0.5 kcal mol^–1^ after depositing 599 Gaussian functions (simulation starting from ^1,4^*B*) and 583 (^4^*C*_1_). The widths of the Gaussians were set to 0.030, 0.040, and 0.015 Å for CV1, CV2, and CV3, respectively, and a Gaussian biasing function was added every 250 MD steps. The widths of the Gaussian functions were taken as half of the standard deviation of a given CV in an unbiased equilibration run, whereas the deposition time was taken as the average time in between five consecutive peaks of CVs, approximately.^40^ Conformational landscapes were explored until the free energy difference between two local minima in both systems (starting from ^4^*C*_1_ or ^1,4^*B*) remains constant (Figure S3D). A total of 909 Gaussian functions (113.63 ps) were deposited in the simulation starting from ^1,4^*B* and 885 functions (110.63 ps) in the one starting from ^4^*C*_1_. All simulations, including the one in the gas phase, resulted in a quantitatively similar conformational FEL (Figure S3A). Only the Northern hemisphere of the Cremer–Pople sphere^20^ was sampled, indicating that Southern hemisphere conformations are of high energy.

The reaction mechanism was explored using QM/MM metadynamics using one CV in the form of a linear combination of distances (*d*) corresponding to all covalent bonds that are expected to be formed/broken during the reaction: *d*(N_δ_–H)_His302_ – *d*(H_His302_–O′) + *d*(C1–O′) – *d*(O_w_–C1) + *d*(O_w_–H_w_) – *d*(H_w_–O_Asp90_) (Figure S4). The Gaussian height was initially set to 1.5 kcal mol^–1^ and was lowered to 0.5 kcal mol^–1^ as the system approached the TS. The Gaussian width and deposition time were set to 0.20 Å and 100 MD steps, respectively. The simulation was stopped after two recrossings over the TS. The first and second recrossings took place once the 897 and 1644 Gaussian functions were deposited (corresponding to 44.85 and 82.2 ps, respectively). The two obtained R → P → R cycles were used to compute the standard deviation and standard error. In the case of the metadynamics simulations starting with the *–1* sugar in a ^4^*C*_1_ conformation, recrossing over TS was not possible due to Asp90/Glu303 competition for the water proton or the system sampling unphysical reaction pathways (Figures S6–S8). Since the free energy barrier usually changes little upon recrossing, the very large free energy barriers obtained from these simulations are indicative of unfeasible reactions.

## Supporting Information Available

Possible conformations of 3,6-anhydro-l-galactose; hydrogen bond interactions of the putative catalytic water; FEL of the *–1* sugar; collective variable components used in the metadynamics simulations; two distinct configurations of the product state; reaction free energy profiles; representative configurations of the QM/MM metadynamics simulation with the *–1* sugar in ^4^*C_1_* ; computed titration curve for His302; RMSD and SASA evolutions; analysis of the Lys260-O’ and the glycosidic bond distances; and pKa values of catalytically relevant residues (PDF)
