# Supplementary material for: An Unusual His/Asp Dyad Operates Catalysis in Agar-Degrading Glycosidases
Source: ACS Catal. 2024 Nov 1;14(22):16897–904. doi: 10.1021/acscatal.4c04139 (PMC11574756; doi:10.1021/acscatal.4c04139)
Supplement: Supplementary file 1 — cs4c04139_si_001.pdf [file cs4c04139_si_001.pdf]

SUPPORTING INFORMATION

An unusual His/Asp dyad operates catalysis in agar-degrading glycosidases.

Mert Sagiroglugil[1], Alba Nin-Hill[1], Elizabeth Ficko-Blean[2], Carme Rovira[1,3,*]

[1]Departament de Química Inorgànica i Orgànica & IQTCUB, Universitat de Barcelona, Martí i Franquès 1, 08028 Barcelona, Spain. [2] CNRS, Sorbonne Université, UMR8227. Laboratory of Integrative Biology of Marine Models, Station Biologique de Roscoff, 29688 Roscoff. France. [3]Institució Catalana de Recerca i Estudis Avançats (ICREA), Passeig Lluís Companys, 23, 08020 Barcelona, Spain.

Corresponding autor e-mail address: c.rovira@ub.edu

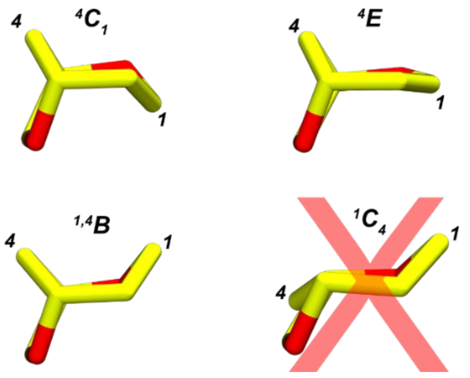

Figure S1. Possible conformations of 3,6-anhydro-L-galactose (isolated molecule).

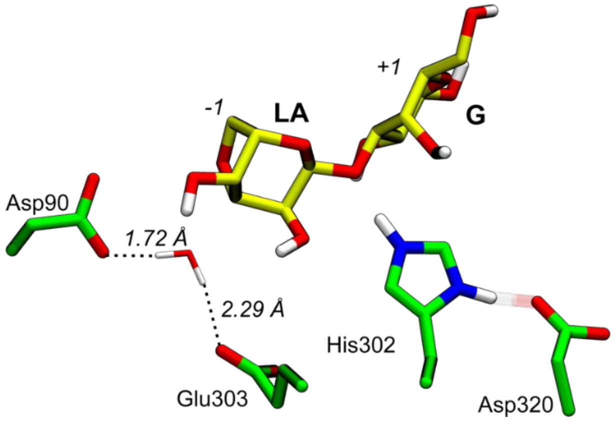

Figure S2. Hydrogen bond interactions of the putative catalytic water in the active site of *Pp*GH117 when the *-1* sugar is in the relaxed $^4C_1$ conformation.

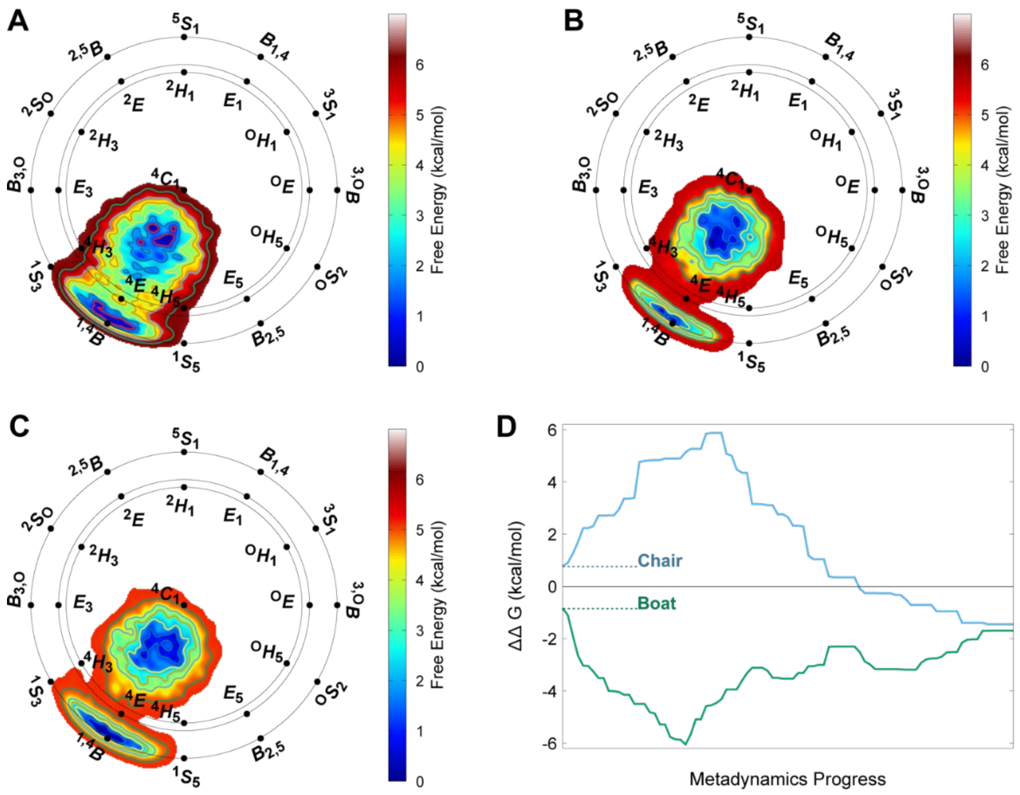

Figure S3. (A) Conformational free energy landscape (FEL) of the *-1* sugar (LA) as an isolated molecule. (B) Conformational FEL of *-1* sugar in the enzyme complex, considering the boat conformation as starting configuration. (C) Conformational FEL of *-1* sugar in the enzyme complex, considering the chair conformation as starting configuration. (D) Convergence of the free energy difference between the two minima of the FEL in (B) and (C).

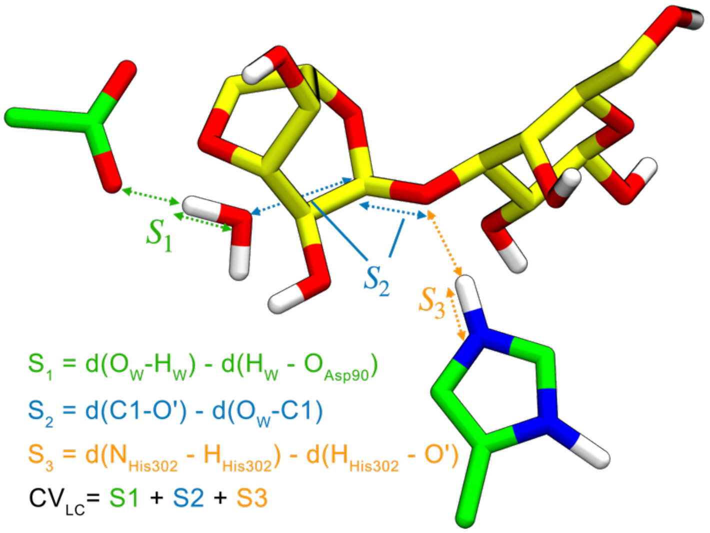

Figure S4. Collective variable components used in the metadynamics simulations of the reaction mechanism.

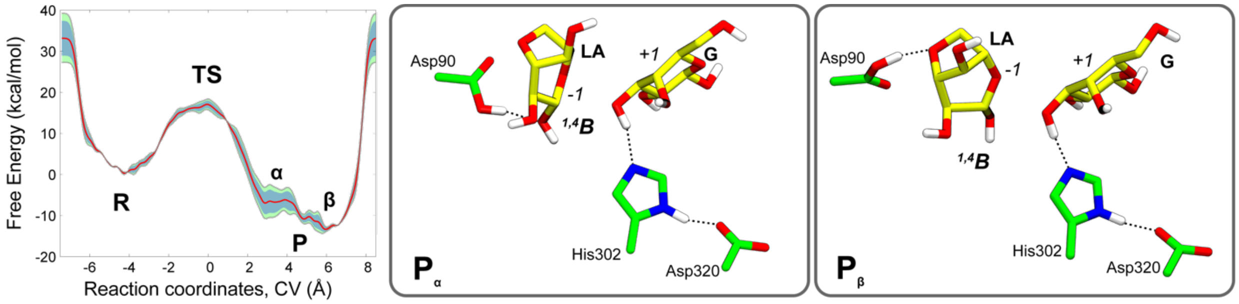

Figure S5. Two distinct configurations (α and β) of the product state. Once the reaction was complete, the system further evolved towards a configuration in which Asp90 rotates to interact with the oxygen of the sugar bicyclic group.

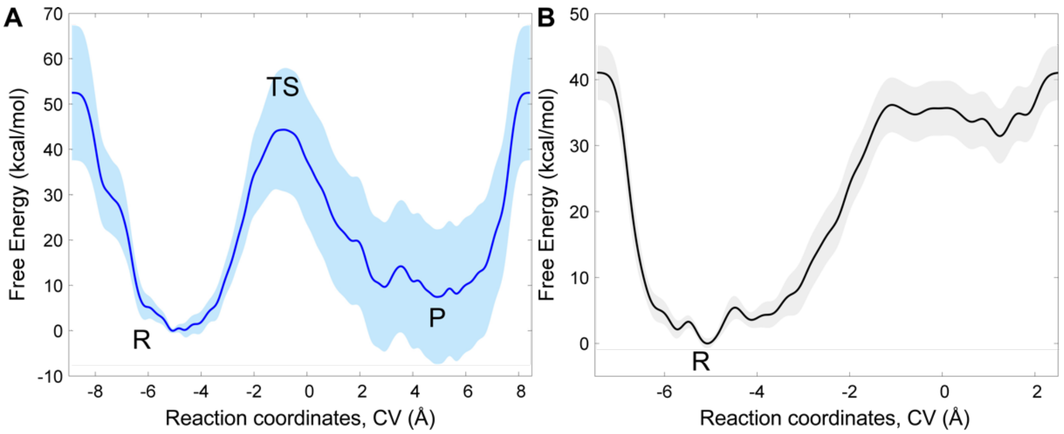

Figure S6. Reaction free energy profiles corresponding to QM/MM metadynamics simulations starting from the *-1* sugar in the $^{4}C_{1}$ conformation. (A) Reaction in which Asp90 acts as the catalytic base. (B) Reaction in which Glu303 acts as a catalytic base. Color filled regions indicate the standard deviation according to the free energy estimator of Tiwary and Parrinello.[1] Changes in the active site along each reaction coordinate are depicted in Figures S7 and S8.

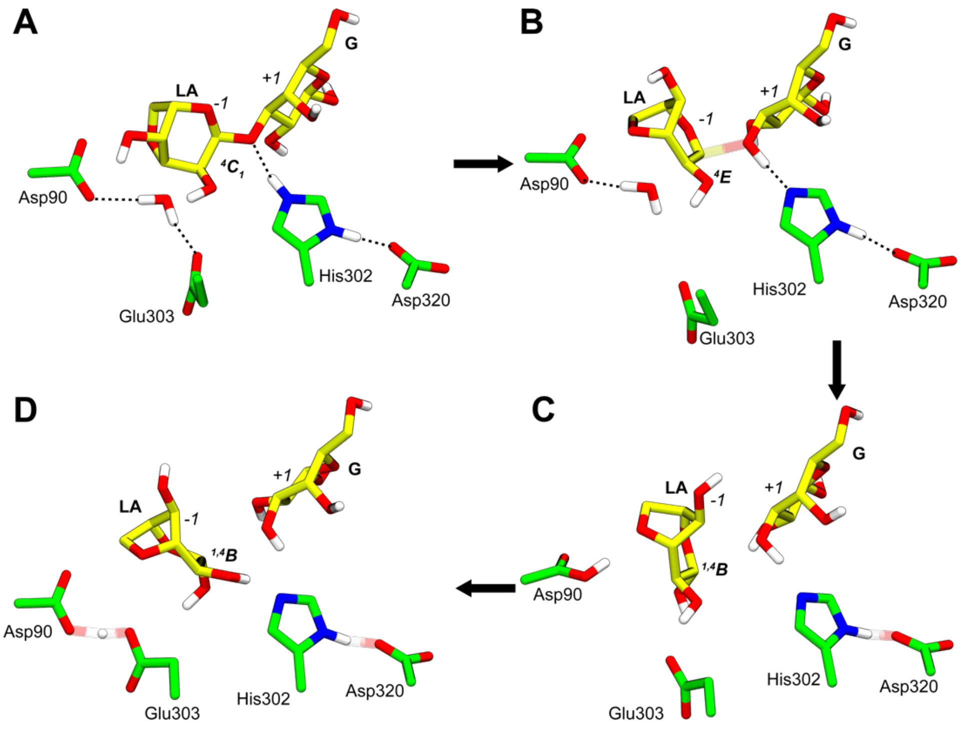

Figure S7. Representative configurations of the QM/MM metadynamics simulation starting from the *-1* sugar in the $^4C_1$ conformation, in which Asp90 is driven as the general base. (A) The reactant state, in which Glu303 is in an orientation similar to with the crystal structure. (B) The transition state, in which the proton is transferred from the His/Asp dyad, elongating the glycosidic bond and the *-1* subsite adopts a ^4E conformation. (C) The product state, in which the Asp90 has deprotonated the catalytic water and the *-1* sugar adopts a $^{1,4}B$ conformation. (D) Another configuration from the product state in which Asp90 and Glu303 compete for the proton. This precludes the reverse reaction sampling necessary to fulfill the metadynamics convergence criteria, resulting in significant uncertainty in the reaction free energy barrier when using the Tiwary-Parrinello scheme[1] (Figure S6, left). Nevertheless, the energy barrier is much higher than the one obtained for the reaction starting with the *-1* sugar in the most stable $^{1,4}B$ conformation (16.8 kcal/mol, manuscript Figure 4A).

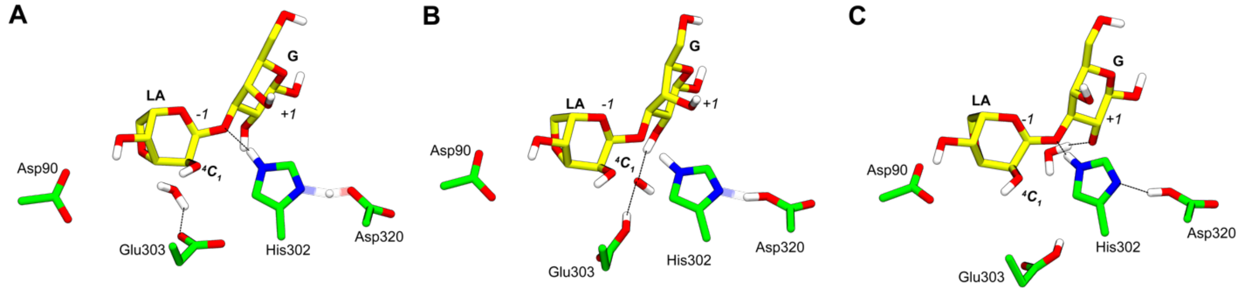

Figure S8. Representative configurations of the QM/MM metadynamics simulation starting from the *-1* sugar in the $^4C_1$ conformation, in which Glu303 is driven as the general base. (A) The reactant state, in which the His/Asp dyad shares a proton and Glu303 is well poised to abstract a proton from the catalytic water. Subsequently, Glu303 deprotonates the water molecule, forming a hydroxide anion. (B) The highest energy configuration along the reaction pathway. (C) High energy product configuration (Figure S6, right), in which the hydroxide anion has taken a proton from the C2-OH of the *+1* sugar, rather than attacking the anomeric carbon of the *-1* sugar.

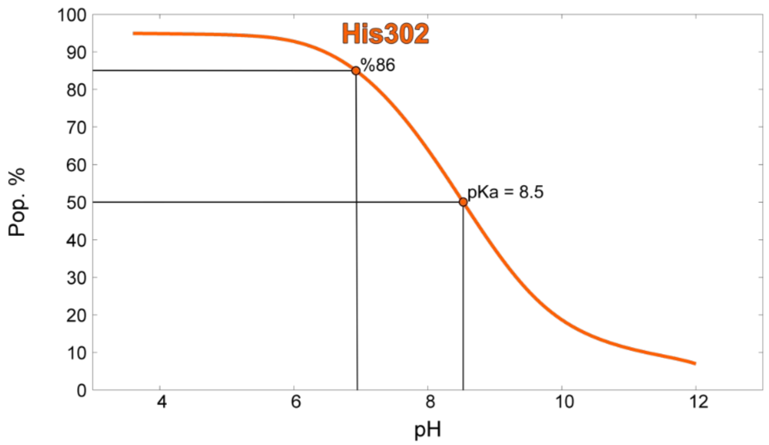

Figure S9. Computed titration curve for His302, indicating the populations of its protonation state at pH 7 (86%) and its pKa value (8.5).

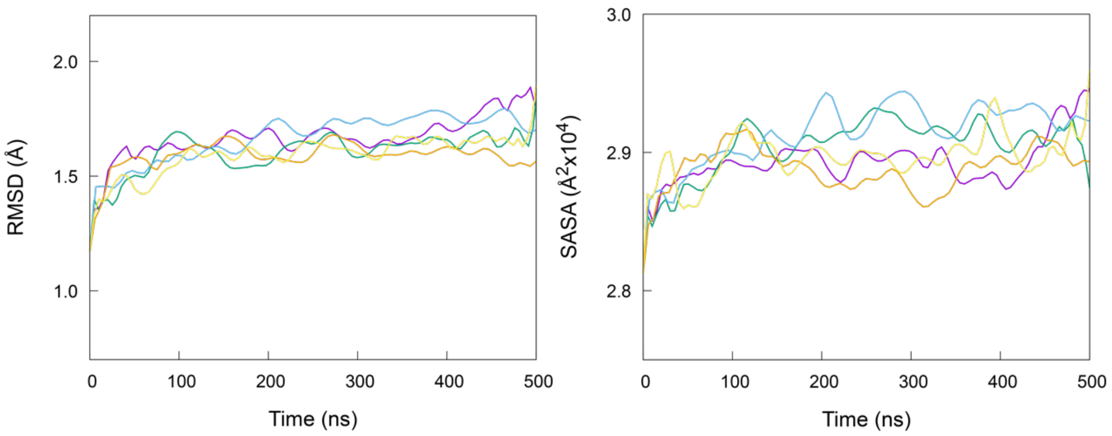

Figure S10. RMSD and SASA evolutions of the enzyme corresponding to a MD simulation in which the conformation of the *-1* sugar is restrained to $^{1,4}B$. Disordered loops from both terminals are truncated.

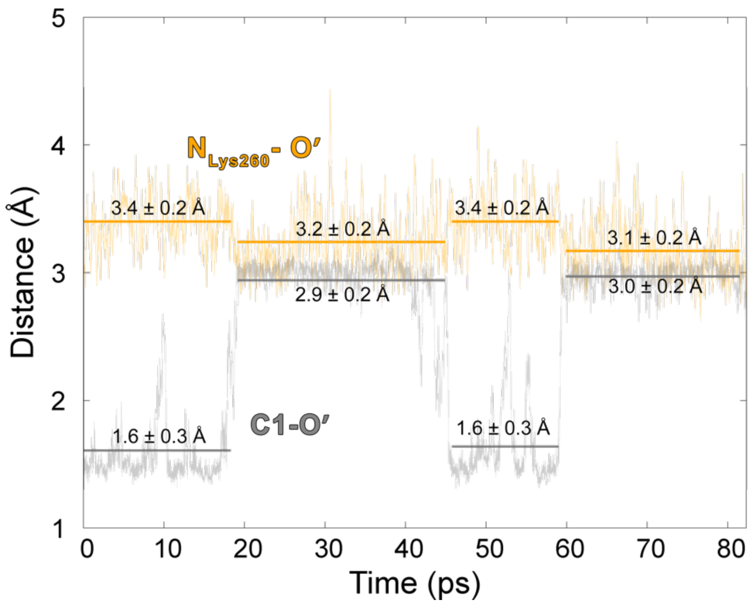

Figure S11. Evolution of the Lys260-O' and the glycosidic bond distances during the QM/MM metadynamics simulation of the chemical reaction.

Table S1. PKa values of catalytically relevant residues, calculated using the H++ server. In case that the titration curve fits into the classical Henderson-Hasselbalch sigmoidal shape, pKas are taken as equal to $pK_{1/2}$, otherwise ($pK_{1/2}$ < 0.0) they are equal to the intrinsic pKa (pKa_{int}).[2]

Residue	pKa_{int}	$pK_{1/2}$
Asp90	5.9	<0.0
His302	5.5	8.5
Glu303	5.8	<0.0
Asp320	6.3	<0.0

References

(1) Tiwary, P.; Parrinello, M. A time-independent free energy estimator for metadynamics. *J. Phys. Chem. B* **2015**, *119*, 736-742.

(2) Onufriev, A.; Case, D. A.; Ullmann, G. M. A Novel View of pH Titration in Biomolecules. *Biochemistry* **2001**, *40*, 3413-3419.
